# Fracture of the Spine

**Published:** 1855-12

**Authors:** 


Fracture of the Spine.—There are two questions which naturally suggest themselves in connection with the treatment of fracture of the spine, with displacement, viz: 1st. Should a reduction of the fracture be attempted? 2d. Should the surgeon, in a case of fracture of the vertebra, followed by displacement and paralysis, proceed to cut through the superincumbent parts at the seat of injury, and endeavor to remove, as has been proposed, any portion of the bone which he may find compressing the spinal cord, and thus treat the case on the same principle as he would a depressed fracture of the skull, accompanied by symptoms of compression of the brain? With regard to the first question, all authorities of weight are, with very few exceptions, agreed on the danger and impropriety of attempting reduction, when the fracture is situated in either the cervical or dorsal regions. In respect of the second question, Heister, a century since, laid it down as a rule of practice, that in the worst cases of fractures of the spine, "the surgeon must lay bare the fractured vertebra with a scalpel, or else remove such fragments as injured the spinal marrow." But it does not appear that either Heister or any of his cotemporaries, as far as we have been able to make out, had recourse to such a proceeding. More recently (in the year 1814), Mr. H. Cline, jr., proposed the operation of trephining or sawing away the vertebral arch, with a view of relieving the compressed spinal cord in fractures of the spine with displacement; and such an operation was not only performed by Mr. Cline, with the utmost hopes of success, but his example was followed by Tyrrell, South, Wickham, Attenburrow, Holschier, Smith, and Rogers. The operation, however, unfortunately proved unsuccessful in every case in which it was attempted. Experience has, therefore, declared against Cline's operation, while pathology is opposed to it, inasmuch as innumerable post-mortem examinations, conducted by impartial observers, have not only proved that in the generality of cases of fractures of the spine, the cord is violently concussed or lacerated, as well as compressed, the usual consequences of which are rapid ramollissement, and subsequently, disorganization; but they have likewise made it manifest that the chief pressure is, in the majority of these cases, not exercised by the vertebral arch, but by the broken body of the lower vertebra projecting backwards, a part obviously, as Mr. Liston has properly remarked, "completely out of the reach of any operation whatever."—(*Dr. Hughes in Dublin Hospital Gazette.*) *Ibid.*

